# High-Throughput Sequencing of Small RNA Transcriptome Reveals Salt Stress Regulated MicroRNAs in Sugarcane

**DOI:** 10.1371/journal.pone.0059423

**Published:** 2013-03-27

**Authors:** Mariana Carnavale Bottino, Sabrina Rosario, Clicia Grativol, Flávia Thiebaut, Cristian Antonio Rojas, Laurent Farrineli, Adriana Silva Hemerly, Paulo Cavalcanti Gomes Ferreira

**Affiliations:** 1 Laboratório de Biologia Molecular de Plantas, Instituto de Bioquímica Médica, Universidade Federal do Rio de Janeiro, Cidade Universitária, Rio de Janeiro, RJ, Brazil; 2 Universidade Federal da Integração Latino-Americana, Foz do Iguaçu, PR, Brazil; 3 Fasteris SA, Plan-les-Ouates, Switzerland; University of Arizona, United States of America

## Abstract

Salt stress is a primary cause of crop losses worldwide, and it has been the subject of intense investigation to unravel the complex mechanisms responsible for salinity tolerance. MicroRNA is implicated in many developmental processes and in responses to various abiotic stresses, playing pivotal roles in plant adaptation. Deep sequencing technology was chosen to determine the small RNA transcriptome of *Saccharum sp* cultivars grown on saline conditions. We constructed four small RNAs libraries prepared from plants grown on hydroponic culture submitted to 170 mM NaCl and harvested after 1 h, 6 hs and 24 hs. Each library was sequenced individually and together generated more than 50 million short reads. Ninety-eight conserved miRNAs and 33 miRNAs* were identified by bioinformatics. Several of the microRNA showed considerable differences of expression in the four libraries. To confirm the results of the bioinformatics-based analysis, we studied the expression of the 10 most abundant miRNAs and 1 miRNA* in plants treated with 170 mM NaCl and in plants with a severe treatment of 340 mM NaCl. The results showed that 11 selected miRNAs had higher expression in samples treated with severe salt treatment compared to the mild one. We also investigated the regulation of the same miRNAs in shoots of four cultivars grown on soil treated with 170 mM NaCl. Cultivars could be grouped according to miRNAs expression in response to salt stress. Furthermore, the majority of the predicted target genes had an inverse regulation with their correspondent microRNAs. The targets encode a wide range of proteins, including transcription factors, metabolic enzymes and genes involved in hormone signaling, probably assisting the plants to develop tolerance to salinity. Our work provides insights into the regulatory functions of miRNAs, thereby expanding our knowledge on potential salt-stressed regulated genes.

## Introduction

Sugarcane *(Saccharum sp.)* has an important role in the world agribusiness, with a central importance in sugar and ethanol production [1]. Grown mainly in tropical countries, sugarcane is a highly polyploid plant, with up to 10 copies of each chromosome, which hinders complete genome sequencing and a thorough genetic, physiologic and biochemical analysis. The commercial sugarcane hybrids are mixtures of two wild parent species, *Saccharum officinarum* and *Saccharum spontaneum*, the result of recent hybridization events [2]. The most important challenge for current global feeding and biofuel production is to satisfy an ever increasing demand of crop productivity in parallel with technological improvements, providing a more sustainable production. The negative impact of abiotic stresses on plant physiology, metabolism and productivity is a major limitation to agriculture. In particular, salinity stress has been regarded as one of the major constraints to increase productivity worldwide [3]. Salinity affects plant growth and development by eliciting sodium toxicity and impairing ionic and osmotic homeostasis [4]. Even though sensors of cell plasma membrane involved in adaptive responses are yet to be identified, it is known that protein phosphorylation/dephosphorylation, Ca^2+^ sensing and changes in phospholipid metabolism are some of the signaling pathways employed by the cell machinery to confer salt tolerance to plants [5], [1]. The effects of salinity on plant species have been studied extensively in several plant species [6], [7], [8], [9], but much less is known about the regulatory mechanisms behind molecular and physiological responses to salt stress in non-model plants, such as sugarcane. Still, some efforts have been made in recent years to unravel the regulatory events controlling the responses to abiotic stresses in sugarcane [10], [11], [12].

Recently, the microRNA and short-interfering RNA mechanisms of action have gained attention because they act as important regulators of gene expression in animals and plants [13], [14]. MiRNAs are small (20–24 nts in length) non-coding RNA derived from longer imperfect hairpin-like structure that contains the mature miRNA and, on the complementary strand, the miRNA* [15], [16]. This pair is subsequently excised by Dicer-like enzymes (DCL1) in the nucleus generating a duplex, miRNA:miRNA*. In the cytoplasm, the mature miRNA is preferentially assembled into the RNA-induced silencing complex (RISC) containing Argonaute1 (AGO1), directing the miRNA to repress its mRNA targets. Initially, it was thought that the miRNA* was rapidly degraded or accumulated at low levels, suggesting a lack of regulatory functionality. However, recent studies have demonstrated that miRNA* accumulates under particular conditions and regulates a different target of that regulated by its cognate mature miRNA [17], [18]. Plant miRNAs change their expression during development or in response to environmental challenges. In plants, microRNA were first reported as targeting genes involved in development, particularly transcription factors such as MYBs, HD-ZIPs, ARFs, GRFs [19], [20]. However, it has been shown that microRNAs can also target other types of genes involved in nutrient transport and metabolic production such as osmolytes, antioxidant enzymes, and transporter proteins [21], [22], [23], [24].

As a first step to increase our understanding of the epigenetic regulation of salt stress tolerance in sugarcane, we carried out deep sequencing of small RNA libraries to identify small RNAs differentially expressed in plantlets grown under salt stress condition in hydroponic cultures. We identified 98 conserved miRNAs, representing 25 families. From these, 11 miRNAs were chosen to evaluate their temporal expression and, for this purpose, we prepared biological replicas. In addition, we set up another experiment where sugarcane plantlets in hydroponic cultures were treated with 340 mM NaCl, a severe saline stress. Roots from both treatments were also harvested. In addition, plants grown from stalks were submitted to mild salt stress to assess the expression of miRNAs on cultivars grown on soil. Finally, putative targets of differentially regulated microRNA were chosen to evaluate whether they had their expression modulated by salt stress. Our results showed differential miRNA expression profiles in response to this abiotic stress, suggesting a fine-tuning regulation in sugarcane. In addition, a set of microRNA and their targets showed inversed pattern of expression in a group of 4 different sugarcane genotypes, indicating that they could be tested as markers of salinity stress. This work is the first report on microRNA regulation in sugarcane plants submitted to salt stress, and it is a useful resource to understand the regulatory role of miRNA in the process of salt tolerance.

## Materials and Methods

### Plant Material and Growth Conditions

For hydroponic cultures, *Saccharum sp* Cv. SP70-1143 plantlets were grown *in vitro* for approximately 1 month in Murashige and Skoog media supplemented with kinetin (0,1 mg/ml), Indol butyric acid (I-BA- 0,2 mg/ml) and citric acid (150 mg/ml). Next, these plantlets were transferred to a hydroponic system supplied with 1× Hoagland’s solution and grown for 2 months [25]. After the acclimation period, salinity stress was induced with a concentration of 170 mM (mild) and 340 mM (severe) NaCl in plantlets by adding the salt to the Hoagland solution. Stalks of 4 cultivars, namely SP83-2847, SP83-5073, SP90-3414 and SP90-1638 (provided by the Centro de Tecnologia Canavieira - CTC) were grown in pots with a mixture of washed sand and soil (2∶1) for approximately 5 months. Plants grown on soil were watered with 170 mM NaCl, and the controls, only with water. The *time-course* established was the same used for the small RNA library construction (0, 1, 6 and 24 hs). For each time point, three biological replicas were submitted to stress, and other three used as controls. Individual plants were used for the analysis. Shoots and roots were collected separately and kept at –80°C until further processing. All experiments were carried in a greenhouse at 28°C, 16 hours/light and 8 hours/dark.

### RNA Extraction, Construction of Small RNA Libraries and Deep Sequencing

Total RNA was extracted with the TRIZOL reagent according to the manufacturer’s instructions (Invitrogen). To validate the salinity stress, the expression profile of the SsNAC23 gene (Figures S1 A, B, C) - described as a marker of saline stress response [26] - was tested in each sample by qPCR using the following primers: F/5′ CGAGAAGACCAACTGGATCA 3′, R/5′ GCCCTCCCTTCTTGTTGTAG 3′). RNA from shoots of plants grown in the hydroponic system submitted to 170 mM NaCl stress (one the biological replicas) were sent to Fasteris Life Sciences SA (Plan-les-Ouates, Switzerland) for small RNA library construction and deep sequencing. Quality Control (QC) was also evaluated to guarantee no contaminations from adapter-self ligation or other non genome-matching sequences. Small RNAs of 18–28 nucleotides were fractionated, isolated and ligated with 5′ and 3′ adapters. They were then used for reverse transcription and subsequent PCR. The final PCR product was purified and sequenced in the Illumina HiSeq100.

### Bioinformatics-based Analysis

After Illumina sequencing generated the reads, computation analysis was performed on the UEA sRNA toolkit-Plant website (http://srna-tools.cmp.uea.ac.uk/plant/cgi-bin/srna-tools.cgi/ [27]. After removal of the 3′ adapter and t/rRNAs sequences, redundant sequences were mapped to the miRNAs deposited in the miRBase (http://www.mirbase.org/) allowing up to 3 mismatches. Using the same bioinformatics platform, we predicted potential targets for sugarcane miRNAs. The *Saccharum officinarum* ESTs – DFCI gene Index release 3 databank (http://compbio.dfci.harvard.edu/tgi/) was used to search for complementary sequences to the miRNAs, allowing no more than three mismatches between miRNA and targets. A list of putative targets is shown in Table 1. To identify differences in miRNAs expression profiles, sequences were computed dividing the number of reads of each sample by the total number of reads of each library, normalized per million (Table S1). MiRNAs were identified with roman numerals due to the large amount of small RNAs generated. Sequences were deposited at GEO (Gene Expression Omnibus)/NCBI with accession number GSE42484. Using a cut-off of 50 reads, the differences of microRNA expression of the conserved miRNAs in the four libraries was analyzed.

**Table 1:** Conserved miRNAs IDs and predicted targets gene families.

sRNA ID	Target gene accession	Start-end position of target	Target description
miR156 V	TC152554	410–429	Squamosa Binding Protein-domain protein 5 (SBP)
miR159 XVI	TC134732	551–568	GAMyb protein
miR166 III	TC139748	868–885	Class III HD-Zip protein 4
miR167 V	TC116192	466–485	Auxin response factor 17 (ARF)
miR168 II	TC114876	146–163	Argonaute 1 (AGO1)
miR169 III	TC140885	528–547	HAP12 - CCAAT-box transcription factor complex
miR396 II	TC126554	333–351	Growth-regulating factor 1 (GRF1)
miR397 III	TC117778	821–841	Laccase-10 precursor (LAC)
miR398 II	TC136123	131–150	Selenium binding protein
miR398* I	TC141387	638–656	Serine/threonine kinase-like protein ABC1063 (Kinase398)
miR528 I	TC149276	143–161	Putative laccase

### Quantitative RT-PCR Assay

To validate the results from the bioinformatics-based analysis, we used the other biological replicas. Plantlets from hydroponic culture treated with 170 mM and 340 mM NaCl, and leaves from stalks grown in soil treated with 170 mM NaCl. Total RNA was prepared as described above. One µg of high quality total RNA was used to synthesize cDNA with Superscript III reverse transcriptase (Invitrogen). Stem-loop Real-time PCR assays [28] were performed with three technical replicas using 1 µl of each cDNA dilution using the SYBR Green Master Mix (Applied Biosystems) in an ABI 7500 sequence detection system. GAPDH and 28S were used as housekeeping genes (GAPDH - F/5′CACGGCCACTGGAAGCA3′, R/5′TCCTCAGGGTTCCTGATGCC3′; 28S: F/5′GCGAAGCCAGAGGAAACT3′, R/5′GACGAACGATTTGCACGTC3′). Specific primers designed for the most abundant miRNAs are listed in Table S2. For analysis of the results, we used the 2^−ΔΔCT^ method as described in [29] to evaluate gene expression. Student’s t-test was used as a statistical tool for the analysis of differences in expression among the triplicates (n = 3, p<0,05).

## Results

### Bioinformatics-based Analysis

**Figure 1:**
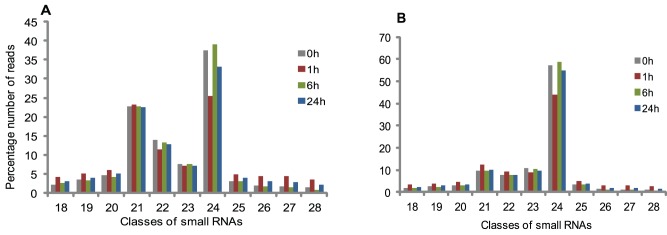
Size distribution of small RNA sequences. Redundant (A) and unique (B) sequences shows that the majority of reads are 21 and 24 nts in length.

Deep sequencing analysis is a powerful strategy that allows the identification and quantification of differences in expression of even low-abundant sRNAs. Small RNA sequence from shoot plantlets generated from Illumina sequencing allows the determination of the abundance of various miRNA families. We constructed four libraries prepared from salinity-stressed sugarcane plants grown in hydroponic culture after 1, 6 and 24 hs of treatment with 170 mM NaCl and from control plants (0 h). We identified a total of 12,622,894, 11,632,867, 12,198,711 and 14,332,005 sequence reads, respectively from 0, 1, 6 and 24 hs totalizing 50,786,477 reads. After trimming the adaptors and filtering tRNAs and rRNAs, 28,017,706 redundant and 12,630,757 unique reads ranging from 18–28 nt were obtained. From these results, the most abundant classes of small RNA found were 21 and 24-nt, which is in agreement with the profiles described in most reports of deep sequencing of plants small RNA libraries (Figure 1) [17], [30], [31], [32]. In the non-redundant fraction, the percentage of 24-nt reads represents the largest proportion, indicating a higher complexity in this class. Matching small RNA sequences to known miRNAs allowed the identification of 25 conserved miRNA families, with 98 individual miRNAs and 33 miRNA* in total (Table S1). Putative new microRNA candidates were also identified and reported elsewhere [33].

**Figure 2:**
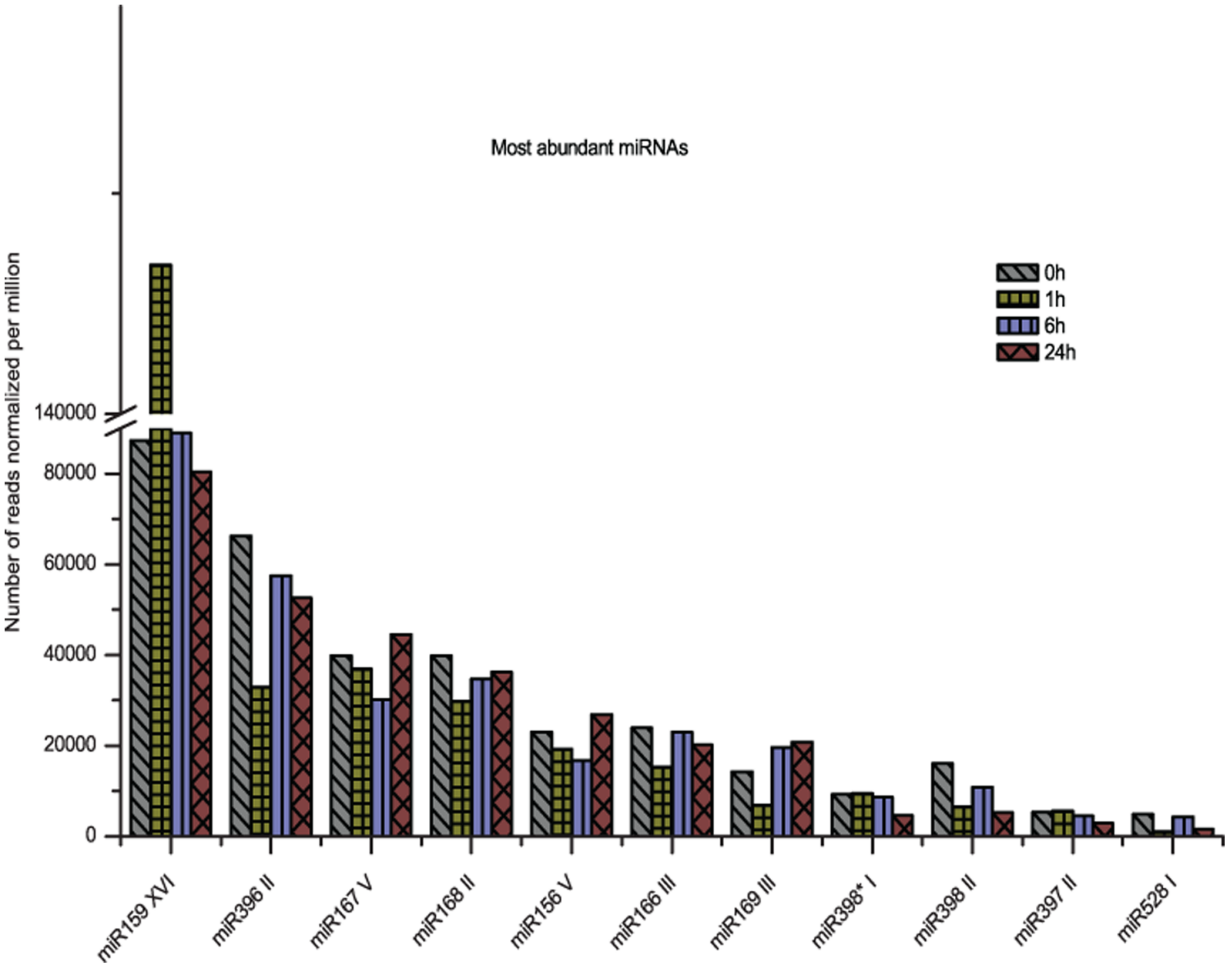
Differential expression profile of most abundant miRNAs obtained in shoots from hydroponic culture. The relative abundance was obtained by the number of reads normalized per million and selected for further validation.

**Figure 3:**
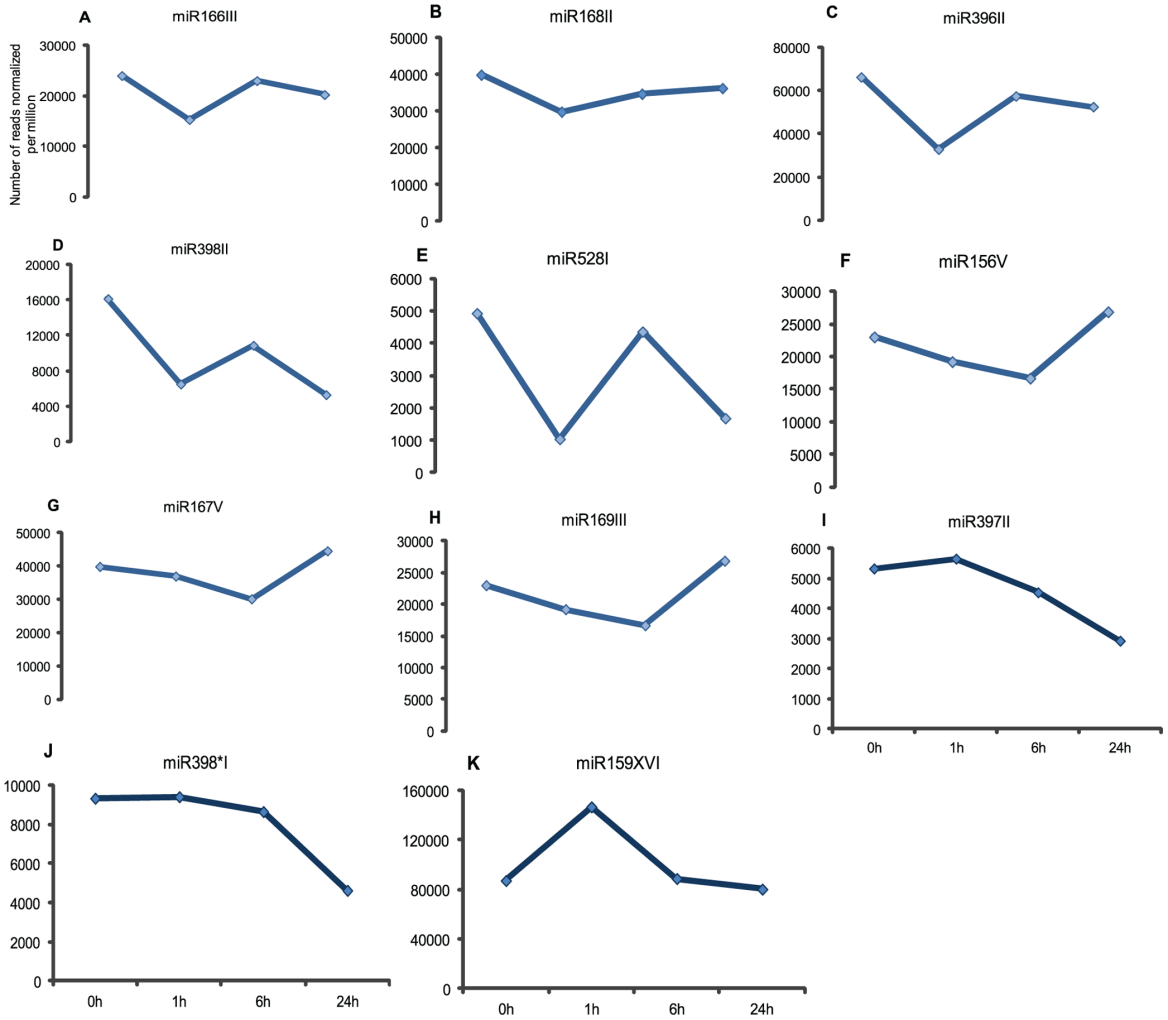
Representative graphs of each miRNA expression profile obtained from samples sent to deep sequencing. A–K: Expression profile of Cv. SP70-1143 shoots grown *in vitro* on 170 mM NaCl. The five categories are: First (A, B, C), second (D, E), third (F, G, H), fourth (I, J) and fifth (K).

The miRNA exhibited variable abundances, with the number of reads ranging from a few hundreds to thousands. Out of these, we highlighted the 10 most abundant miRNA and one microRNA398* in the four libraries, which were selected for validation in biological replicas (Figure 2). microRNA*398 was selected because it was the most abundant microRNA* present in the libraries. Based on the expression patterns observed, the microRNAs were clustered in five categories to facilitate our comprehension regarding their modulation in response to salt stress. The first cluster comprises 3 miRNAs; miR166III, 168III and 396II (Figures 3A–C), which were down-regulated at 1 h and then returned to their original levels at 6 hs, and down-regulated again at 24 hs; the second group comprises miR398II and 528I (Figures 3D–E), which were rapidly repressed at 1 h, then up-regulated at 6 hs and then down-regulated again after 24 hs; the third group, which includes miR156V, 167V and 169III (Figures 3F–H), was down-regulated until 6 hs and then up-regulated at 24 hs; in the fourth group, miR397II and 398*I (Figures 3I–J) were repressed at 6 hs and 24 hs; finally, miR159XVI (Figure 3K) showed a specific expression profile being quickly induced at 1 h of stress and repressed after that.

### Analysis of Stress-responsive microRNAs in Shoots and Roots Submitted to Mild and Severe Salt Treatments

**Figure 4:**
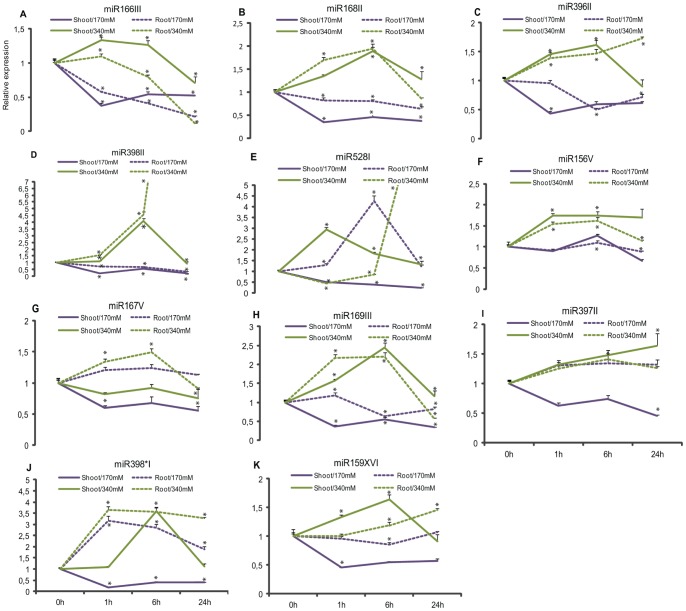
Validation of the effects of salinity stress on miRNA expression. A–K represents the relative expression of shoots and roots of Cv. SP70-1143 of biological replicas submitted to 170 mM and 340 mM NaCl treatment in hydroponic system. Bars indicate means ± standard deviation. Significant differences of expression are indicated with asterisk.

Stem-loop qRT-PCR was performed in biological replicas to analyze the expression of the 11 selected sRNA. MiRNA 166III, 168II, 396II and 398II had similar expression profiles in the small RNA libraries and in the shoot biological replicas (Figures 3A–D, Figures 4A–D). In contrast, miRNAs 528I, 156V, 167V, 169III, 397II, 398*I and 159XVI had expression patterns that did not match the profiles of the small RNA libraries (Figures 3E–K, Figures 4E–K). The differences in expression observed among the samples that were sequenced and the ones used for validation could be the result of either physiological differences among samples or high fluctuation of miRNA expression when plants are under stress [34].

In the roots, miR166III, 168II, 396II, 398II, 169III, and 159XVI were down-regulated (Figures 4A, B, C, D, H, K), with the expression of miR166III being strongly suppressed. On the other hand, miRNAs 528I, 167V, 397II and 398*I showed induction at 6 hs in response to moderate salt stress in the roots (Figures 4E, G, I, J). In this group, we observed that 398*I showed a 3-fold increase in expression in 1 h, and miR528I, a 4-fold increase after 6 hs.

When plants were submitted to a severe salinity stress, miRNAs 166III, 168II, 396II, 398II, 156V, 169III, 397II and 159XVI showed a 1,5-fold increase in expression (Figures 4A, B, C, D, F, H, I, K). MiR528I showed a 3-fold induction in shoots, and miR398*I, a 4-fold induction in roots (Figures 4E, J, respectively). In general, both tissues displayed a similar expression modulation of miRNAs under these conditions; miRNAs were induced in 1 h and maintained their level until 6 hs, decaying after that. However, miR397II was continually induced in shoots and miR398II in roots/340 mM. In view of these results, miRNAs had higher expression rates in samples treated with severe salt treatment compared to the mild one.

### Analysis of microRNA Expression in Leaves From Soil-grown Sugarcane Stalks

**Figure 5:**
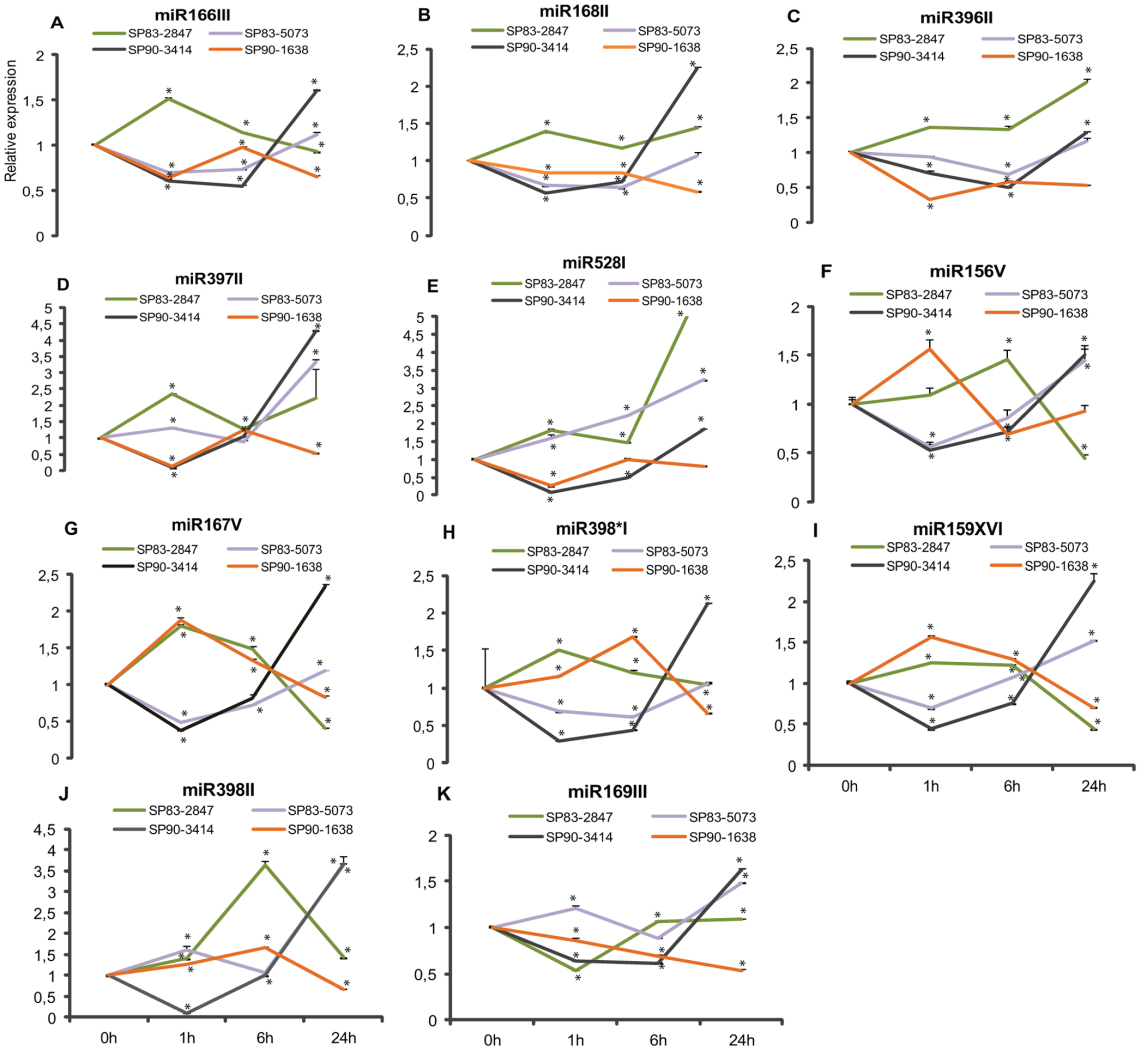
Verification of miRNA profile in plants grown from stalks. A–K represents the expression of the previously selected 11 miRNAs in shoots of the genotypes (SP90-1638, SP90-3414, SP83-2847 and SP83-5073). Bars indicate means ± standard deviation. Significant differences of expression are indicated with asterisk.

The analysis of miRNA regulation in plants germinated from stalks and grown in pots with soil is important because the plant’s settings resemble its physiological conditions found in the field. To investigate the responsiveness of miRNA to salinity in shoots from grown stalks, we compared the expression profiles of the same chosen group of 11 sRNA in shoots from four cultivars (SP83-2847, SP83-5073, SP90-3414 and SP90-1638) irrigated with 170 mM NaCl (Figures 5A–K).

Most cultivars could be grouped according to their expression response to stress as follows: miRNAs 166III, 168II, 396II, 397II and 528I showed a common response in the four cultivars, despite being more expressed in SP83-2847. In the latter cultivar, the miRNAs were induced at 1 h, returning to the initial level at 6 hs and induced at 24 hs (Figures 5A, B, C, D, E). Conversely, in the other three cultivars, these miRNAs were repressed at 1 h followed by an induction at 6 hs and; however, at 24 hs we observed more variation in the expression patterns.

A group including miRNAs 156V, 167V, 398*I and 159XVI also displayed common patterns of expression. Figures 5F, G, H and I indicate that in SP90-1638 and SP83-2847 these miRNAs were more expressed when compared to their levels in SP90-3414 and SP83-5073. In the first two cultivars, these miRNAs were induced at 1 h and then repressed at 6 and 24 hs, except for the induction of miRNA398*I observed at 6 h in SP90-1638 (Figures 5H). In contrast, in the latter two cultivars, a repression occurred at 1 h and 6 hs, followed by an up-regulation in 24 hs.

The miR398II was repressed at 1 h and induced after 6 hs only in SP90-3414. In contrast, its expression was induced at 1 h and repressed after 6 hs in SP90-1638 and SP83-2847 (Figures 5J). SP83-5073 also showed an induction at 1 h, but it was repressed at 6 hs and induced at 24 hs, which is unlike the pattern described above. Still in this cultivar, miR169III showed a similar regulation compared to miR398II (Figures 5K). However, miR169III levels were down-regulated in 1 h in SP83-2847, SP90-3414 and SP90-1638, but in the next time-points, the modulation varied.

### Regulation of Putative microRNA Targets in Salt-stressed Plants

A crucial step to understand the biological functions of miRNAs is the identification of their targets and the analysis of their regulation. Plant miRNAs use to the near-perfect sequence complementarity to a target mRNA as a mechanism of post-transcriptional regulation. The resulting cleavage of targets leads to a decrease of mRNA levels. To better understand the functionality of the 10 sRNAs analyzed in this work, putative targets were predicted using the UEA sRNA toolkit-Plant website with the *Saccharum officinarum* ESTs – DFCI gene Index allowing no more than 3 mismatches between the miRNA and the target. In Table 1 the putative targets identified are listed, with their respective gene accession numbers.

We performed qRT-PCR of six chosen targets to evaluate if the miRNA differential expression observed had a direct consequence in their target transcript abundance in biological replicas and in the cultivars. The mRNA-targets identified are mainly transcription factors involved in plant development, such as GAMyB (miR159XVI), HAP12 (miR169III) and GRF (miR396II), as well as the Argonaute 1 (AGO1) protein, important for directing the cleavage of miRNAs’ targets. We also predicted some enzymes that might be involved plant metabolism: Laccase (miR397III) and Ser/Thr kinase protein (miR398*I).

**Figure 6:**
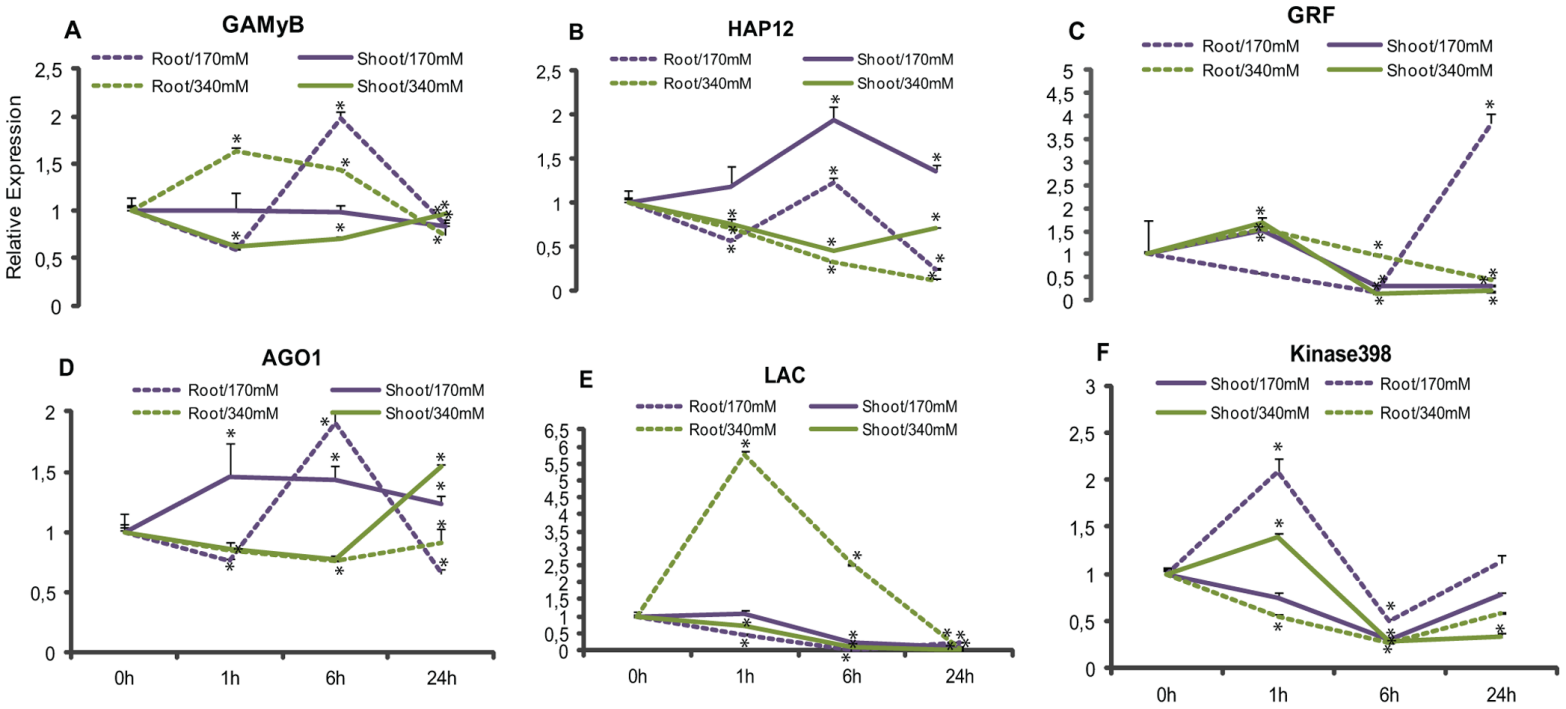
Evaluation of target expression profile of shoots and roots from hydroponic culture. Validation of target regulation by miRNA in roots and shoots of Cv SP70-1143 submitted to 170 mM and 340 mM NaCl treatments. Bars indicate means ± standard deviation. Significant differences of expression are indicated with asterisk.

As shown in figures 4 and 6, target miRNA-mediated regulation appears to be occurring, but not at all time points. It is possible that expression levels of target mRNA does not decrease with miRNA induction suggesting that mRNA post-transcriptional regulation is not limited to miRNA-direct cleavage [35]. Previously, the GAMyB transcription factor had been identified as a target of miR159 [36], [37]. Figure 6A shows that GAMyB levels are higher in roots under mild and severe salt stress. Interestingly, expression levels in shoots remained relatively constant through time when compared to miR159XVI expression profile in that same tissue.

Another gene that was identified as responsive to salt stress was the transcription factor HAP, also known as Nuclear Factor Y (NF-Y). Figure 6B shows the expression profiles of HAP12 in samples submitted to mild and severe salt treatments. In the moderate stress, mRNA levels in roots was down-regulated in 1 h and up-regulated in shoots, followed by an increase in 6 hs and decrease in 24 hs, which is inversely correlated to the miR169III expression (Figure 4H, Figure 6B). In the severe condition, roots and shoots were equally repressed until 6 hs, followed by an induction in shoots, while in roots the mRNA levels are continuously low (Figure 6B).

Growth-regulating factors (GRF) constitute a family of transcription factors involved in growth and development of plants. The results of the expression analysis showed that GRF1 was likely regulated by miR396II (Figure 4C, Figure 6C). In roots grown in 170 mM NaCl, GRF mRNA levels started to drop within 1 h of salt stress, while miR396II level did not change. However, after 6 hs, the target expression increased 4-fold compared to the miRNA (Figure 6C). The induction of GRF genes occurred after 1 h in shoots and roots from the severe salt treatment.

Previous works have described the feedback regulation of AGO1 mRNA through the action of miR168 as being a crucial step for proper plant development and regulation of gene expression [38], [39]. Here, we have shown that AGO1 was highly induced in tissues of plants grown on mild salt concentration (Figure 6D). AGO1 levels were induced after 1 h in shoots and maintained a constant level till the end of the experiment. In roots, a 2-fold induction was observed after 6 hs followed by a down-regulation in 24 hs. In the severe salt treatment, lower expression levels of AGO1 were observed in root and leaf tissues, compared to miR168II levels in the same salt concentration.

Laccases have also been identified as miR397 targets in other plants [40], [41]. They are part of a larger group of enzymes, termed the multi-copper enzymes, which also includes the ascorbate oxidase. Here, the experiment showed a 6-fold increase in Laccase expression in roots treated with 340 mM NaCl after in 1 h compared to miR397II (Figure 4I, Figure 6E). The level of Laccase transcripts in shoots from both salt treatments, as well as in roots treated with 170 mM NaCl seemed unaffected.

Recent reports suggested the contribution of miRNA* on the regulation of gene expression under particular environmental conditions [42], [18]. We identified a Serine/Threonine protein kinase (named here as Kinase398) as a putative miR398*I target. In samples from the mild stress, Kinase398 mRNA levels in roots showed a 2-fold induction in 1 h, and then these levels were down-regulated until 6 hs. In shoots, Kinase398 was repressed after 1 h (Figure 6F). In shoots grown on severe salt stress, Kinase398 expression showed a similar pattern from the observed in roots grown in170 mM NaCl, being down-regulated until 6 hs (Figure 6F).

### Target Analysis in Plants Germinated from Stalks

**Figure 7:**
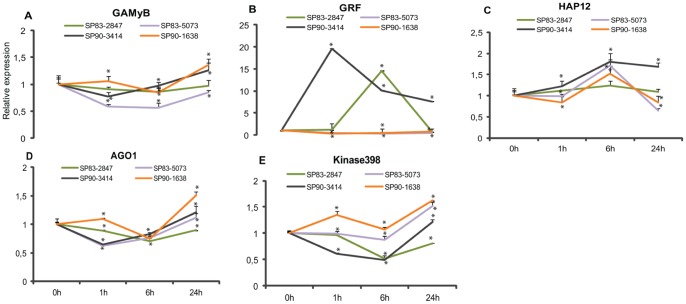
Targets’ analysis in shoot of plants germinated from stalks. Expression profile of the predicted targets in shoots in response to mild salt stress in genotypes SP90-1638, SP90-3414, SP83-2847 and SP83-5073. Bars indicate means ± standard deviation. Significant differences of expression are indicated with asterisk.

Next, we conducted an analysis of the same miRNA targets in plants grown from stalks. The analysis of HAP12 and GRF expression confirmed that the targets had an inverse regulation from that of miRNAs 169III and 396II, respectively, in the set of four cultivars (Figure 7A, Figure 7B). Our results showed an approximately 2-fold induction of HAP12 in shoots and roots treated with 170 mM NaCl, and in the four genotypes. In contrast, a continuous decrease in mRNA levels occurred in roots and shoots treated with 340 mMNaCl. GRF mRNA levels were down-regulated after 1 h in sugarcane plantlets, and, in shoots of SP90-1638 and SP83-5073, it remained constant through the time-course. In contrast, GRF was up-regulated in SP83-2847 and SP90-3414, differently from the pattern observed in the others genotypes.

GAMyB, AGO1 and Kinase398 expression modulation showed the expected inverse profile relative to their respective miRNA in cultivars SP83-2847 and SP90-1638. However, this result was not observed in cultivars SP83-5073 and SP90-3414 suggesting that these targets might not be directly involved in miRNA gene expression in response to salt treatment in these cultivars.

## Discussion

Plant metabolism and physiological integrity are affected by salt stress. Yet, many plants are capable to tolerate alterations in their surrounding environment by developing a more efficient management of osmotic adjustment and compartmentalization of solutes [43]. In this work, we identified millions of small RNA sequences of sugarcane shoot plantlets as a starting point to understand the extent of the impact of salt stress on plant metabolism through the regulation by miRNAs. Our results demonstrated that the expression of miRNAs and their predicted targets in sugarcane are modulated by salt stress.

Short-term stress applied in the experiments described here was important to analyze how the earliest responses are perceived by the plant in a saline environment. Bioinformatics-based analysis identified miRNA 166III, 168III, 396II, 398II, 528I, 156V, 167V and 169III as repressed already after the first hour of exposure to salt, whereas miR397II and 398*I expression levels were affected by salt stress only after 6 hs. A different pattern from the described above was observed for miR159XVI, whose increase in transcription levels was initiated in 1 h. This result demonstrates that these miRNAs are responsive to salt stress, but are differentially regulated at specific time-points after the stress. Other reports that emphasized the rapid plant response to an abiotic stress also observed various patterns of expression. Liu et al. [19] showed by microarray that two-week-old seedlings of *Arabidopsis* grown under 300 mM NaCl, miRNAs 156, 167 and 168 demonstrated a gradual increase of expression after 2 h, while miR396a increased its level after 24 h. The authors suggested that these miRNAs might be of later response or were not sensitive enough to perceive the salt stressed imposed to *Arabidopsis* seedlings.

To validate the results obtained by deep sequencing, we constructed biological replicas with plantlets grown in hydroponic culture, and harvested shoots and roots treated with 170 mM NaCl. To investigate the modulation of miRNAs responses to a more severe stress, plantlets were treated with 340 mM NaCl for 1, 6 and 24 hours. Compared to control plants, it was possible to observe an interesting regulation of miRNAs in treated plants in both conditions, which reflected in the different patterns of expression. Most of the miRNAs seemed to be induced when plants were treated with a more severe salt stress rather than with the mild one (Figure 4). In plants treated with 340 mM NaCl, miRNAs 166III, 168II, 396II, 398II, 156V, 169III and 159XVI showed higher expression rates in shoots and roots compared to the samples from the mild stress treatment (Figures 4A, B, C, D, F, H, K). Shoots and roots are obligated to deal with excess of Na^+^ and Cl^-^ and to quickly adjust to changes in gene expression. The reason for the lower level of expression in the mild stress could be explained by the fact that this salt concentration was not enough to cause physiological changes in the plant that would trigger a regulation mediated by miRNA.

MiR167V, 397II and 398*I showed higher expression rates in roots independent of the NaCl concentration applied, suggesting that they might operate more actively in this tissue (Figures 4G, I, J). The role of miR398*I has not yet been described, although our results indicated an important correlation of this miRNA* and salt stress in sugarcane. MiR528I presented a varied expression in tissues influenced by the same salt concentration. It was induced in shoots, but repressed in roots treated with 340 mM NaCl; and, repressed in shoots and induced in roots under 170 mM NaCl. In [44], the authors showed that miR528 transcripts were more expressed in leaves of *Saccharum sp*, and Ding et al. [36] reported that osa-miR528 was regulated by in salt-stressed in roots of two maize lines. They also predicted Cu/Zn superoxide dismutase as a miR528 target and they associated this enzyme to the scavenging of reactive oxygen species, produced by salt stress. Here, we predicted a Laccase target instead. Several lines of evidence report the involvement of Laccases in lignin synthesis in cell wall and also in plant growth and development [45]. One possibility is that the 6-fold induction of Laccase in salt stressed plants would lead to the formation of Casparian strips located in sugarcane plantlets root cells. Theses strips are formed by the deposition of lignin and suberin [46] contributing, not only to a strengthening of the cell wall, but also acting as a barrier to solutes preventing the influx of salt ions, and the efflux of vital solutes [47].

A better understanding of the roles of miRNAs and their targets is of fundamental importance to trace their biochemical function in order to decode the complex biological network that they might be part of. Overall, qRT-PCR of the chosen targets matched the inverse expected correlation of expression of miRNAs, thus suggesting that these targets are involved in salt stress responses. Positively regulated miRNAs are expected to influence the expression of negative regulators of stress tolerance by repressing them. On the other hand, negatively regulated miRNAs allows the induction of positive stress regulators [48]. Transcription factors validated in our work, such as GAMyB, HAP12 and GRF, are known to regulate the development and growth of plants, and these targets had already been reported in previous works as being modulated by salt stress [36], [37], [49], [50].

Individual HAP subunits are known to be required in important processes, such as embryogenesis, drought resistance, ABA perception, nitrogen-fixing nodule development, among others [51], [52], [53]. Zhao et al. [8] described that two members of miR169 family were induced by high salinity in rice and *Arabidopsis* seedlings. In our work, HAP12 expression was induced in shoots of plants germinated from stalks and also in plantlets of mild salt stress. However, the repression observed in roots and shoots treated with 340 mM NaCl could indicate an important regulatory tolerance mechanism displayed by HAP12. GRFs form a family of transcription factors that regulate plant growth and are strongly expressed in actively growing and developing tissues, such as shoot apex, flower buds and immature leaves, but weakly expressed in mature leaves and organs [54], [55]. Under salt stress, GRF expression in the 5 sugarcane genotypes analyzed here showed an opposed pattern of regulation to miR396II. The kinetics of the transcription factors expression varies in the cultivars, suggesting that there could be genetic factors controlling their expression, indicating an important control of plant development, especially when the plants were under the severe salt stress.

Although several AGO proteins have been shown to be regulated by biotic stresses [56], [57], their correlation with abiotic stress responses is still unclear. In our results, it is likely that miR168II transcription is activated in conditions with high salt concentration and, as a consequence, AGO1 levels decay. Since Argonaute is a core component of the RISC complex that guides miRNAs to cleave complementary target-mRNAs, it may impact global miRNA action potentially adjusting metabolic processes in response to salt stress.

Also, we report a novel sugarcane kinase, named Kinase398, and its possible regulation by miRNA398* upon salt stress. An extensive search in the databases did not identified orthologs of the Kinase398 in other plant species. Curiously, we found a sugarcane homologous gene (TC128481) encoding a similar protein kinase, indicating a possible gene duplication (Figure S2). However, the alignment with microRNA398* showed that this gene is not a target (Figure S2B). We suggest that Kinase398 and its homologous gene may be the result of recent evolution, and that Kinase398 may have recently acquired a microRNA-based mode of regulation.

### Conclusion

Here, we have shown that several microRNAs can play important roles in the response to salinity in sugarcane cultivars. MiRNAs were dynamically and transiently regulated during salt stress in the five sugarcane genotypes analyzed. We also showed that target protein-coding genes are stress-regulated as well, and might be indirectly involved in repairing cell wall, establishing metabolic adjustments and regulating development and cell division by changing the expression of other proteins that are part of a major regulatory network, yet to be fully elucidated. Together, our results also indicated that an intrinsic regulation of plant growth and development is being modulated during salt stress and it also highlights differences in the nature of the molecular responses from *in vitro* plantlets and plants grown from stalks. Based on our findings, we can conclude that a number of miRNAs are involved in salt stress responses in sugarcane. The observation that microRNAs and targets have patterns of responses to salt stresses that are characteristic of different sugarcane cultivars suggest that they could be used to develop molecular markers, useful in sugarcane breeding. Our findings will probably lead to new ways to enhance crop tolerance to this abiotic problem in agribusiness.

## Supporting Information

Figure S1 **Validation with the SsNAC23 gene**. Two biological replicas confirm the regulation mediated by salinity stress. (A) Shoot samples of Cv. SP70-1143 grown on hydroponics and treated with 170 mM NaCl that were sent to deep sequencing show a tendency in expression in the four-time points; (B) Biological replicas of *in vitro* plantlets grown on 170 mM and 340 mM NaCl; (C) and shoots of four genotypes of plants grown from stalks watered with 170 Mm NaCl. Bars indicate means ± standard deviation. Significant differences of expression are indicated with asterisk.
(EPS)

Figure S2 CLUSTALW alignment between each Kinase398 homologs with miR398*I. (A) Near-perfect complementarity between miR398*I and TC141387, identified by bioinformatics; (B) Despite the fact that TC128481 was identified as a homologous gene, the pairing with the miRNA is discontinuous indicating that it is not a target of miR398I*. *denotes conserved nucleotides.
(EPS)

Table S1 List of miRNAs and miRNAs* IDs and the normalized number of reads per million generated from Solexa sequencing of shoot of plants harvested after 1, 6 and 24 **h in 170** **mM NaCl of hydroponic solution, and the control plants.**
(DOC)

Table S2 **miRNAs and targets primers sequences for RT-PCR analysis.**
(DOC)
