# Supplementary material for: High-Throughput Sequencing of Small RNA Transcriptome Reveals Salt Stress Regulated MicroRNAs in Sugarcane
Source: PLoS One. 2013 Mar 27;8(3):e59423. doi: 10.1371/journal.pone.0059423 (PMC3609749; doi:10.1371/journal.pone.0059423)
Supplement: Table S1 — List of miRNAs and miRNAs* IDs and the normalized number of reads per million generated from Solexa sequencing of shoot of plants harvested after 1, 6 and 24 h in 170 mM NaCl of hydroponic solution, and the control plants. (DOC) [file pone.0059423.s003.doc]

Table S1: List of miRNAs and miRNAs* IDs and the normalized number of reads per million generated from Solexa sequencing of shoot of plants harvested after 1, 6 and 24h in 170mM NaCl of hydroponic solution, and the control plants.

| **Library 0h** | | | **Library 1h** | | | **Library 6h** | | | **Library 24h** | | |
| --- | --- | --- | --- | --- | --- | --- | --- | --- | --- | --- | --- |
| miRNAs ID | Sequences | Norm. reads/  million | miRNAs ID | Sequences | Norm. reads/  million | miRNAs ID | Sequences | Norm. reads/  million | miRNAs ID | Sequences | Norm. reads/  million |
| | miR156 V | | --- | | miR156 VII | | miR156 II | | miR156 IV | | miR156 VI | | miR156 IX | | miR156 XXI | | miR156 XX | | miR156 XVII | | miR156 XIII | | miR156 I | | miR156* I | | miR156* II | | miR156* III | | miR156* IV | | miR156* V | | miR156* VI | | miR156* VII | | miR156* VIII | | miR156* IX | | miR159 XVI | | miR159 XIII | | miR159 X | | miR159 XV | | miR159 XVII | | miR159 XX | | miR159 VIII | | miR159 XIV | | miR160 I | | miR160 II | | miR162 I | | miR164 I | | miR166 III | | miR166 X | | miR166 IX | | miR166 I | | miR166 II | | miR166 XV | | miR166 XVI | | miR166* XIII | | miR166* XI | | miR166* X | | miR166* I | | miR166* XIV | | miR167 V | | miR167 IV | | miR167 VII | | miR167 III | | miR167 IX | | miR167* III | | miR167* II | | miR167* I | | miR168 II | | miR168 XVI | | miR168 I | | miR168 IV | | miR168;miR168* II | | miR169 III | | miR169 V | | miR169 II | | miR169 VIII | | miR169 IX | | miR169 XVIII | | miR169 XVII | | miR169 IV | | miR169 XV | | miR169* V | | miR169* XV | | miR169* VII | | miR171 I | | miR171 XVII | | miR171 XVIII | | miR171 VII | | miR171* II | | miR172 I | | miR172* | | miR319 XII | | miR319 I | | miR319 III | | miR390 I | | miR393 II | | miR393 IV | | miR393 III | | miR393; I | | miR393; II | | miR393;III | | miR394 I | | miR395 III | | miR395 VII | | miR395 I | | miR395 IX | | miR395* I | | miR396 II | | miR396 III | | miR396 XIII | | miR396 VII | | miR396 X | | miR396 VI | | miR396 XIV | | miR396 IX | | miR396 XV | | miR396 IV | | miR396 XVI | | miR396 XVII | | miR396* I | | miR397 II | | miR397 VI | | miR398 II | | miR398 I | | miR398 III | | miR398 IV | | miR398* I | | miR398* II | | miR398* III | | miR398* IV | | miR398* V | | miR398* VI | | miR398* VII | | miR408 I | | miR408 II | | miR408 III | | miR444 III | | miR444 II | | miR5072 I | | miR5072 II | | miR528 I | | miR528 II | | miR528* I | | miR529 I | | miR529* I | | miR827 | | | TGACAGAAGAGAGTGAGCAC | | --- | | TTGACAGAAGAGAGTGAGCAC | | TGACAGAAGAGAGCGAGCAC | | TGACAGAAGAGAGTGAGCA | | TTGACAGAAGAGAGCGAGCAC | | TGACAGAAGAGAGTGAGCACA | | TGACAGAAGAGAGTGAGCACG | | TGACAGAAGAGAGTGAGC | | GACAGAAGAGAGTGAGCA | | CTGACAGAAGAGAGTGAGCAC | | GACAGAAGAGAGTGAGCACA | | GCTCACTTCTCTTTCTGTCAGC | | GCTCACTCTCTATCTGTCAGC | | GCTCACTTCTCTCTCTGTCAGC | | TGCTCACTTCTCTTTCTGTCAGC | | GCTCGCTTCTCTTTCTGTCAGC | | GCTCACTCTCTATCTGTCAG | | GCTCACTTCTCTTTCTGTCAG | | TGCTCACTTCTCTTTCTGTCAG | | TGCTCGCTTCTCTTTCTGTCAGC | | TTTGGATTGAAGGGAGCTCTG | | TTTGGATTGAAGGGAGCT | | TTGGATTGAAGGGAGCTCTG | | TTTGGATTGAAGGGAGCTCT | | TTTGGATTGAAGGGAGCTCTGC | | TTTGCATGACCGAGGAGCTGC | | TTGGATTGAAGGGAGCTCC | | TTTGGATTGAAGGGAGCTC | | TGCCTGGCTCCCTGTATGCCA | | TGCCTGGCTCCCTGAATGCCA | | TCGATAAACCTCTGCATCCAG | | TGGAGAAGCAGGGCACGTGCA | | TCGGACCAGGCTTCATTCCCC | | TCGGACCAGGCTTCATTCCTC | | TCGGACCAGGCTTCATTCCC | | TCTCGGACCAGGCTTCATTCC | | TTCGGACCAGGCTTCATTCCC | | TCGGACCAGGCTTCAATCCCT | | CTCGGACCAGGCTTCATTCCC | | GGAATGTTGTCTGGTTCAAGG | | GGAATGTTGTCTGGCTCGGGG | | GGAATGTTGTCTGGCTCGAGG | | AATGTTGTCTGGCTCGAGGTG | | GGAATGTTGTCTGGTTCTAGG | | TGAAGCTGCCAGCATGATCTGA | | TGAAGCTGCCAGCATGATCTG | | TGAAGCTGCCAGCATGATCTGG | | TGAAGCTGCCAGCATGATCTA | | AAGCTGCCAGCATGATCTGA | | AGGTCATGCTGTAGTTTCATC | | AGATCATCTGGCAGTTTCATT | | AGATCATGTTGCAGCTTCACT | | TCGCTTGGTGCAGATCGGGAC | | TCGCTTGGTGCAGATCGGGA | | CCCGCCTTGCACCAAGTGAAT | | TCGCTTGGTGCAGATCGGGACC | | CCCGCCTTGCACCAAGTGA | | CAGCCAAGGATGACTTGCCGG | | AGCCAAGGATGACTTGCCGG | | CAGCCAAGGATGACTTGCCGA | | AGCCAAGGATGACTTGCCGAT | | AGCCAAGGATGACTTGCCGGC | | TAGCCAAGGATGATTTGCCTGT | | TAGCCAAGGATGAGCTGCCTG | | TAGCCAAGGATGACTTGCCGG | | TAGCCAAGGATGACTTGCCTACA | | TGGCATCCATTCTTGGCTAAG | | GCATCCATTCTTGGCTAAGTG | | TGGGCAGTCTCCTTGGCTAGC | | TGATTGAGCCGTGCCAATATC | | TTGAGCCGCGTCAATATCTCC | | TTGAGCCGTGCCAATATCACG | | TGAGCCGAACCAATATCACTC | | TGTTGGCTCGGCTCACTCAGA | | AGAATCTTGATGATGCTGCAT | | GCAGCACCATCAAGATTCACA | | TTGGACTGAAGGGTGCTCCCT | | CTTGGACTGAAGGGTGCTCCCT | | TTGGACTGAAGGGTGCTCCCTT | | AAGCTCAGGAGGGATAGCGCC | | CTCCAAAGGGATCGCATTGAT | | TCCAAAGGGATCGCATTGAT | | TCCAAAGGGATCGCATTGATC | | TCAGTGCAATCCCTTTGGAAT | | TCAGTGCAATCCCTTTGGAATT | | CAGTGCAATCCCTTTGGAATT | | TTGGCATTCTGTCCACCTCC | | TGAAGTGTTTGGGGGAACTC | | TGAAGTGCTTGGGGGAACTC | | AGTGAAGTGTTTGGGGGAACT | | TGAAGTGTTTGGGGGAACT | | GTTCTCCACAAACTACTTCAGT | | TCCACAGGCTTTCTTGAACTG | | TTCCACAGCTTTCTTGAACTT | | TCCACAGGCTTTCTTGAACT | | CTTTCCACAGGCTTTCTTGAA | | GTTCAAGAAAGTCCTTGGAAA | | ATGGTTCAAGAAAGTCCTTGGAAA | | TTCCACAGCTTTCTTGAACT | | GTTCAAGAAAGCCCATGGAAA | | TTTCCACAGCTTTCTTGAACT | | TTCCACAGCTTTCTTGAACTG | | ACAGGCTTTCTTGAACTG | | GTTCAAGAAAGTCCTTGGAA | | GGTCAAGAAAGCTGTGGGAAG | | TTGAGTGCAGCGTTGATGAGC | | TTGAGTGCAGCGTTGATGAG | | TGTGTTCTCAGGTCGCCCCCG | | ATGTGTTCTCAGGTCGCCCCCG | | GTGTTCTCAGGTCGCCCCCGC | | ATGTGTTCTCAGGTCGCCCCC | | GGGGGCGGACTGGGAACACAT | | GGGGCGGACTGGGAACACATG | | GGGGCGGACTGGGAACACAT | | GGGGCGGACTGGGAACACA | | GGGGGCGGACTGGGAACACA | | GGGGGCGGACTGGGAACAC | | GGGGGCGGACTGGGAACACATG | | CTGCACTGCCTCTTCCCTGGC | | TGCACTGCCTCTTCCCTGGCT | | TGCACTGCCTCTTCCCTGGC | | TGCAGTTGTTGTCTCAAGCTT | | TGCAGTTGTTGCCTCAAGCTT | | TTCCCCAGCGGAGTCGCCA | | GTCCCCAGCGGAGTCGCCA | | TGGAAGGGGCATGCAGAGGAG | | TGGAAGGGGCATGCAGAGGA | | CCTGTGCCTGCCTCTTCCATT | | AGAAGAGAGAGAGTACAGCCT | | GCTGTACCCTCTCTCTTCTTC | | TTAGATGACCATCAGCAAACA | | | 23018,60 | | --- | | 1868,12 | | 570,38 | | 559,91 | | 212,58 | | 135,40 | | 96,81 | | 96,81 | | 81,76 | | 81,11 | | 67,37 | | 9967,90 | | 9133,92 | | 1894,94 | | 542,25 | | 401,62 | | 283,23 | | 256,41 | | 90,92 | | 67,37 | | 87309,91 | | 2881,99 | | 1461,92 | | 1256,54 | | 233,52 | | 85,69 | | 72,61 | | 65,41 | | 869,31 | | 130,82 | | 407,51 | | 451,33 | | 23995,18 | | 4235,33 | | 1065,54 | | 860,80 | | 681,58 | | 520,01 | | 67,37 | | 475,53 | | 231,55 | | 116,43 | | 67,37 | | 67,37 | | 39886,66 | | 4894,01 | | 2600,07 | | 1353,34 | | 165,49 | | 300,23 | | 166,80 | | 98,12 | | 39899,74 | | 12457,43 | | 758,76 | | 540,94 | | 89,61 | | 14253,60 | | 310,05 | | 297,62 | | 265,57 | | 121,66 | | 115,78 | | 99,42 | | 94,19 | | 91,57 | | 315,93 | | 266,87 | | 208,01 | | 650,83 | | 296,96 | | 122,32 | | 66,06 | | 126,24 | | 131,48 | | 75,88 | | 3787,92 | | 185,77 | | 90,27 | | 86,34 | | 2204,99 | | 117,74 | | 78,49 | | 201,46 | | 87,65 | | 87,65 | | 272,76 | | 2570,63 | | 111,20 | | 77,84 | | 65,41 | | 104,66 | | 66244,46 | | 3074,95 | | 729,98 | | 557,95 | | 305,47 | | 279,30 | | 131,48 | | 121,66 | | 107,27 | | 96,15 | | 96,15 | | 67,37 | | 165,49 | | 5326,38 | | 105,31 | | 16144,61 | | 790,16 | | 398,35 | | 103,35 | | 9335,38 | | 3299,96 | | 281,27 | | 228,28 | | 226,32 | | 75,88 | | 71,95 | | 2212,18 | | 1576,39 | | 541,60 | | 94,19 | | 75,22 | | 388,54 | | 189,04 | | 4928,68 | | 243,98 | | 190,34 | | 778,38 | | 471,61 | | 248,56 | | | miR156 V | | --- | | miR156 VII | | miR156 IV | | miR156 II | | miR156 VI | | miR156 XVII | | miR156 XX | | miR156 IX | | miR156 XXI | | miR156 XIII | | miR156* I | | miR156* II | | miR156* III | | miR156* IV | | miR156* VII | | miR156* VI | | miR156* V | | miR159 XVI | | miR159 XIII | | miR159 XV | | miR159 X | | miR159 XVII | | miR159 XIV | | miR159 XX | | miR160 I | | miR162 I | | miR164 I | | miR166 III | | miR166 X | | miR166 IX | | miR166 I | | miR166 XV | | miR166 II | | miR167 V | | miR167 IV | | miR167 VII | | miR167 III | | miR167* III | | miR167* II | | miR168 II | | miR168 XVI | | miR168 I | | miR168 IV | | miR168;miR168* II | | miR169 III | | miR169 II | | miR169 VIII | | miR169 V | | miR169 XVII | | miR169* VII | | miR169* V | | miR171 I | | miR171 XVII | | miR171 XVIII | | miR319 XII | | miR319 I | | miR393 II | | miR393; I | | miR394 I | | miR395 III | | miR395 | | miR396 II | | miR396 III | | miR396 XIII | | miR396 VII | | miR396 VI | | miR397 II | | miR398 II | | miR398 III | | miR398 I | | miR398 V | | miR398 IV | | miR398* I | | miR398* V | | miR398* IV | | miR398* II | | miR398* III | | miR398* VI | | miR398* VII | | miR408 I | | miR408 II | | miR408 III | | miR444 III | | miR444 II | | miR5072 I | | miR5072 II | | miR528 I | | miR528 II | | miR529 I | | miR529* I | | miR827 | | | TGACAGAAGAGAGTGAGCAC | | --- | | TTGACAGAAGAGAGTGAGCAC | | TGACAGAAGAGAGTGAGCA | | TGACAGAAGAGAGCGAGCAC | | TTGACAGAAGAGAGCGAGCAC | | GACAGAAGAGAGTGAGCA | | TGACAGAAGAGAGTGAGC | | TGACAGAAGAGAGTGAGCACA | | TGACAGAAGAGAGTGAGCACG | | CTGACAGAAGAGAGTGAGCAC | | GCTCACTTCTCTTTCTGTCAGC | | GCTCACTCTCTATCTGTCAGC | | GCTCACTTCTCTCTCTGTCAGC | | TGCTCACTTCTCTTTCTGTCAGC | | GCTCACTTCTCTTTCTGTCAG | | GCTCACTCTCTATCTGTCAG | | GCTCGCTTCTCTTTCTGTCAGC | | TTTGGATTGAAGGGAGCTCTG | | TTTGGATTGAAGGGAGCT | | TTTGGATTGAAGGGAGCTCT | | TTGGATTGAAGGGAGCTCTG | | TTTGGATTGAAGGGAGCTCTGC | | TTTGGATTGAAGGGAGCTC | | TTTGCATGACCGAGGAGCTGC | | TGCCTGGCTCCCTGTATGCCA | | TCGATAAACCTCTGCATCCAG | | TGGAGAAGCAGGGCACGTGCA | | TCGGACCAGGCTTCATTCCCC | | TCGGACCAGGCTTCATTCCTC | | TCGGACCAGGCTTCATTCCC | | TCTCGGACCAGGCTTCATTCC | | TCGGACCAGGCTTCAATCCCT | | TTCGGACCAGGCTTCATTCCC | | TGAAGCTGCCAGCATGATCTGA | | TGAAGCTGCCAGCATGATCTG | | TGAAGCTGCCAGCATGATCTGG | | TGAAGCTGCCAGCATGATCTA | | AGGTCATGCTGTAGTTTCATC | | AGATCATCTGGCAGTTTCATT | | TCGCTTGGTGCAGATCGGGAC | | TCGCTTGGTGCAGATCGGGA | | CCCGCCTTGCACCAAGTGAAT | | TCGCTTGGTGCAGATCGGGACC | | CCCGCCTTGCACCAAGTGA | | CAGCCAAGGATGACTTGCCGG | | CAGCCAAGGATGACTTGCCGA | | AGCCAAGGATGACTTGCCGAT | | AGCCAAGGATGACTTGCCGG | | TAGCCAAGGATGAGCTGCCTG | | TGGGCAGTCTCCTTGGCTAGC | | TGGCATCCATTCTTGGCTAAG | | TGATTGAGCCGTGCCAATATC | | TTGAGCCGCGTCAATATCTCC | | TTGAGCCGTGCCAATATCACG | | TTGGACTGAAGGGTGCTCCCT | | CTTGGACTGAAGGGTGCTCCCT | | CTCCAAAGGGATCGCATTGAT | | TCAGTGCAATCCCTTTGGAAT | | TTGGCATTCTGTCCACCTCC | | TGAAGTGTTTGGGGGAACTC | | TGAAGTGTTTGGAGGAACTC | | TCCACAGGCTTTCTTGAACTG | | TTCCACAGCTTTCTTGAACTT | | TCCACAGGCTTTCTTGAACT | | CTTTCCACAGGCTTTCTTGAA | | ATGGTTCAAGAAAGTCCTTGGAAA | | TTGAGTGCAGCGTTGATGAGC | | TGTGTTCTCAGGTCGCCCCCG | | GTGTTCTCAGGTCGCCCCCGC | | ATGTGTTCTCAGGTCGCCCCCG | | GTGTTCTCAGGTCGCCCCCG | | ATGTGTTCTCAGGTCGCCCCC | | GGGGGCGGACTGGGAACACAT | | GGGGGCGGACTGGGAACACA | | GGGGCGGACTGGGAACACA | | GGGGCGGACTGGGAACACATG | | GGGGCGGACTGGGAACACAT | | GGGGGCGGACTGGGAACAC | | GGGGGCGGACTGGGAACA | | CTGCACTGCCTCTTCCCTGGC | | TGCACTGCCTCTTCCCTGGCT | | TGCACTGCCTCTTCCCTGGC | | TGCAGTTGTTGTCTCAAGCTT | | TGCAGTTGTTGCCTCAAGCTT | | TTCCCCAGCGGAGTCGCCA | | GTCCCCAGCGGAGTCGCCA | | TGGAAGGGGCATGCAGAGGAG | | TGGAAGGGGCATGCAGAGGA | | AGAAGAGAGAGAGTACAGCCT | | GCTGTACCCTCTCTCTTCTTC | | TTAGATGACCATCAGCAAACA | | | 19232,14 | | --- | | 1542,31 | | 1046,65 | | 442,58 | | 171,20 | | 160,74 | | 108,40 | | 91,96 | | 84,48 | | 75,51 | | 4844,48 | | 4626,93 | | 1437,64 | | 392,49 | | 183,16 | | 140,55 | | 113,64 | | 146761,56 | | 3847,92 | | 2363,93 | | 2169,55 | | 462,77 | | 178,68 | | 101,67 | | 571,17 | | 129,34 | | 145,04 | | 15294,50 | | 2360,94 | | 577,90 | | 554,72 | | 225,78 | | 179,43 | | 36981,02 | | 2145,63 | | 1245,51 | | 696,77 | | 124,10 | | 85,23 | | 29821,21 | | 8610,91 | | 682,56 | | 343,90 | | 130,08 | | 6943,01 | | 297,55 | | 282,59 | | 122,61 | | 101,67 | | 265,40 | | 142,04 | | 666,86 | | 196,62 | | 82,24 | | 536,03 | | 76,26 | | 1280,65 | | 100,18 | | 358,10 | | 1840,60 | | 142,79 | | 32903,59 | | 1116,17 | | 396,98 | | 280,35 | | 139,80 | | 5652,64 | | 6506,41 | | 808,16 | | 623,50 | | 151,02 | | 121,86 | | 9414,59 | | 937,50 | | 302,03 | | 169,71 | | 167,46 | | 153,26 | | 74,76 | | 2180,02 | | 1667,91 | | 586,12 | | 172,70 | | 103,92 | | 449,31 | | 208,58 | | 1044,40 | | 313,25 | | 2650,26 | | 1910,88 | | 287,08 | | | miR156 V | | --- | | miR156 VII | | miR156 II | | miR156 IV | | miR156 VI | | miR156 XXI | | miR156 XX | | miR156 IX | | miR156* I | | miR156* II | | miR156* III | | miR156* IV | | miR156* V | | miR156* VII | | miR156* VI | | miR156* VIII | | miR159 XVI | | miR159 XIII | | miR159 X | | miR159 XV | | miR159 XVII | | miR159 XIV | | miR159 VIII | | miR160 I | | miR160 II | | miR162 I | | miR164 I | | miR166 III | | miR166 X | | miR166 IX | | miR166 I | | miR166 II | | miR166 XV | | miR166* XIII | | miR166* XI | | miR166* X | | miR167 V | | miR167 IV | | miR167 VII | | miR167 III | | miR167 IX | | miR167* III | | miR167* II | | miR168 II | | miR168 XVI | | miR168 I | | miR168 IV | | miR169 III | | miR169 II | | miR169 VIII | | miR169 V | | miR169 XVII | | miR169 XV | | miR169 IX | | miR169* VII | | miR169* V | | miR169* XV | | miR171 I | | miR171 XVII | | miR171 XVIII | | miR172 I | | miR319 XII | | miR319 I | | miR319 III | | miR393 II | | miR393 IV | | miR393; I | | miR393; II | | miR393;III | | miR394 I | | miR395 III | | miR395 | | miR395 VII | | miR395 I | | miR396 II | | miR396 III | | miR396 XIII | | miR396 VII | | miR396 VI | | miR396 X | | miR396 XVI | | miR396 XIV | | miR396 IV | | miR396 XIV | | miR396* I | | miR397 II | | miR398 II | | miR398 III | | miR398 I | | miR398 IV | | miR398* I | | miR398* II | | miR398* V | | miR398* IV | | miR398* III | | miR398* VI | | miR408 I | | miR408 II | | miR408 III | | miR444 III | | miR444 II | | miR5072 I | | miR5072 II | | miR528 I | | miR528 II | | miR528* I | | miR529 I | | miR529* I | | miR827 | | | TGACAGAAGAGAGTGAGCAC | | --- | | TTGACAGAAGAGAGTGAGCAC | | TGACAGAAGAGAGCGAGCAC | | TGACAGAAGAGAGTGAGCA | | TTGACAGAAGAGAGCGAGCAC | | TGACAGAAGAGAGTGAGCACG | | TGACAGAAGAGAGTGAGC | | TGACAGAAGAGAGTGAGCACA | | GCTCACTTCTCTTTCTGTCAGC | | GCTCACTCTCTATCTGTCAGC | | GCTCACTTCTCTCTCTGTCAGC | | TGCTCACTTCTCTTTCTGTCAGC | | GCTCGCTTCTCTTTCTGTCAGC | | GCTCACTTCTCTTTCTGTCAG | | GCTCACTCTCTATCTGTCAG | | TGCTCACTTCTCTTTCTGTCAG | | TTTGGATTGAAGGGAGCTCTG | | TTTGGATTGAAGGGAGCT | | TTGGATTGAAGGGAGCTCTG | | TTTGGATTGAAGGGAGCTCT | | TTTGGATTGAAGGGAGCTCTGC | | TTTGGATTGAAGGGAGCTC | | TTGGATTGAAGGGAGCTCC | | TGCCTGGCTCCCTGTATGCCA | | TGCCTGGCTCCCTGAATGCCA | | TCGATAAACCTCTGCATCCAG | | TGGAGAAGCAGGGCACGTGCA | | TCGGACCAGGCTTCATTCCCC | | TCGGACCAGGCTTCATTCCTC | | TCGGACCAGGCTTCATTCCC | | TCTCGGACCAGGCTTCATTCC | | TTCGGACCAGGCTTCATTCCC | | TCGGACCAGGCTTCAATCCCT | | GGAATGTTGTCTGGTTCAAGG | | GGAATGTTGTCTGGCTCGGGG | | GGAATGTTGTCTGGCTCGAGG | | TGAAGCTGCCAGCATGATCTGA | | TGAAGCTGCCAGCATGATCTG | | TGAAGCTGCCAGCATGATCTGG | | TGAAGCTGCCAGCATGATCTA | | AAGCTGCCAGCATGATCTGA | | AGGTCATGCTGTAGTTTCATC | | AGATCATCTGGCAGTTTCATT | | TCGCTTGGTGCAGATCGGGAC | | TCGCTTGGTGCAGATCGGGA | | CCCGCCTTGCACCAAGTGAAT | | TCGCTTGGTGCAGATCGGGACC | | CAGCCAAGGATGACTTGCCGG | | CAGCCAAGGATGACTTGCCGA | | AGCCAAGGATGACTTGCCGAT | | AGCCAAGGATGACTTGCCGG | | TAGCCAAGGATGAGCTGCCTG | | TAGCCAAGGATGACTTGCCTACA | | AGCCAAGGATGACTTGCCGGC | | TGGGCAGTCTCCTTGGCTAGC | | TGGCATCCATTCTTGGCTAAG | | GCATCCATTCTTGGCTAAGTG | | TGATTGAGCCGTGCCAATATC | | TTGAGCCGCGTCAATATCTCC | | TTGAGCCGTGCCAATATCACG | | AGAATCTTGATGATGCTGCAT | | TTGGACTGAAGGGTGCTCCCT | | CTTGGACTGAAGGGTGCTCCCT | | TTGGACTGAAGGGTGCTCCCTT | | CTCCAAAGGGATCGCATTGAT | | TCCAAAGGGATCGCATTGAT | | TCAGTGCAATCCCTTTGGAAT | | TCAGTGCAATCCCTTTGGAATT | | CAGTGCAATCCCTTTGGAATT | | TTGGCATTCTGTCCACCTCC | | TGAAGTGTTTGGGGGAACTC | | TGAAGTGTTTGGAGGAACTC | | TGAAGTGCTTGGGGGAACTC | | AGTGAAGTGTTTGGGGGAACT | | TCCACAGGCTTTCTTGAACTG | | TTCCACAGCTTTCTTGAACTT | | TCCACAGGCTTTCTTGAACT | | CTTTCCACAGGCTTTCTTGAA | | ATGGTTCAAGAAAGTCCTTGGAAA | | GTTCAAGAAAGTCCTTGGAAA | | ACAGGCTTTCTTGAACTG | | TTCCACAGCTTTCTTGAACT | | TTCCACAGCTTTCTTGAACTG | | TTTCCACAGCTTTCTTGAACT | | GGTCAAGAAAGCTGTGGGAAG | | TTGAGTGCAGCGTTGATGAGC | | TGTGTTCTCAGGTCGCCCCCG | | GTGTTCTCAGGTCGCCCCCGC | | ATGTGTTCTCAGGTCGCCCCCG | | ATGTGTTCTCAGGTCGCCCCC | | GGGGGCGGACTGGGAACACAT | | GGGGCGGACTGGGAACACATG | | GGGGGCGGACTGGGAACACA | | GGGGCGGACTGGGAACACA | | GGGGCGGACTGGGAACACAT | | GGGGGCGGACTGGGAACAC | | CTGCACTGCCTCTTCCCTGGC | | TGCACTGCCTCTTCCCTGGCT | | TGCACTGCCTCTTCCCTGGC | | TGCAGTTGTTGTCTCAAGCTT | | TGCAGTTGTTGCCTCAAGCTT | | TTCCCCAGCGGAGTCGCCA | | GTCCCCAGCGGAGTCGCCA | | TGGAAGGGGCATGCAGAGGAG | | TGGAAGGGGCATGCAGAGGA | | CCTGTGCCTGCCTCTTCCATT | | AGAAGAGAGAGAGTACAGCCT | | GCTGTACCCTCTCTCTTCTTC | | TTAGATGACCATCAGCAAACA | | | 16696,17 | | --- | | 1623,97 | | 527,18 | | 407,34 | | 186,50 | | 93,59 | | 78,10 | | 70,70 | | 6715,36 | | 4136,00 | | 1026,09 | | 402,63 | | 282,78 | | 136,68 | | 96,28 | | 70,02 | | 88920,32 | | 2876,95 | | 1496,04 | | 1327,05 | | 261,24 | | 73,39 | | 68,68 | | 973,57 | | 98,30 | | 385,12 | | 436,96 | | 22989,38 | | 3633,73 | | 910,96 | | 872,58 | | 787,75 | | 487,46 | | 363,57 | | 147,45 | | 144,08 | | 30146,42 | | 5023,39 | | 2517,42 | | 1061,10 | | 150,14 | | 354,15 | | 133,31 | | 34695,15 | | 9296,75 | | 737,92 | | 375,02 | | 19618,91 | | 402,63 | | 375,02 | | 187,17 | | 115,81 | | 114,46 | | 105,71 | | 344,05 | | 317,12 | | 177,75 | | 816,02 | | 256,52 | | 107,73 | | 90,22 | | 3538,12 | | 92,24 | | 74,06 | | 1714,19 | | 79,45 | | 179,77 | | 84,83 | | 79,45 | | 377,71 | | 2852,04 | | 498,23 | | 147,45 | | 86,18 | | 57472,44 | | 2412,39 | | 577,01 | | 558,15 | | 167,65 | | 109,07 | | 103,01 | | 92,24 | | 76,75 | | 69,35 | | 121,19 | | 4533,91 | | 10875,60 | | 565,56 | | 528,53 | | 138,02 | | 8654,43 | | 1873,76 | | 300,96 | | 173,71 | | 167,65 | | 86,18 | | 2223,19 | | 1498,74 | | 588,45 | | 158,22 | | 104,36 | | 368,96 | | 193,91 | | 4359,53 | | 195,25 | | 164,28 | | 1045,61 | | 506,99 | | 249,79 | | | miR156 V | | --- | | miR156 VII | | miR156 IV | | miR156 II | | miR156 VI | | miR156 XXI | | miR156 XVII | | miR156 XX | | miR156 IX | | miR156 XIII | | miR156 I | | miR156* I | | miR156* II | | miR156* III | | miR156* IV | | miR156* V | | miR156* VI | | miR156* VII | | miR159 XVI | | miR159 XIII | | miR159 XV | | miR159 X | | miR159 XVII | | miR159 XIV | | miR160 I | | miR160 II | | miR162 I | | miR164 I | | miR164 II | | miR166 III | | miR166 X | | miR166 I | | miR166 IX | | miR166 II | | miR166 XV | | miR166* X | | miR166* XIII | | miR166* XI | | miR167 V | | miR167 IV | | miR167 VII | | miR167 III | | miR167 IX | | miR167 X | | miR167* III | | miR167* II | | miR168 II | | miR168 XVI | | miR168 I | | miR168 IV | | miR168 V | | miR169 III | | miR169 II | | miR169 VIII | | miR169 V | | miR169 XV | | miR169 IX | | miR169 XVII | | miR169 IV | | miR169* VII | | miR169* V | | miR169* XV | | miR171 I | | miR171 XVII | | miR171 VII | | miR171 XVIII | | miR172 I | | miR319 XII | | miR393 II | | miR393 IV | | miR393; I | | miR394 I | | miR395 III | | miR395 | | miR396 II | | miR396 III | | miR396 XIII | | miR396 VII | | miR396 VI | | miR396 XVI | | miR396 XIV | | miR396 X | | miR396 XV | | miR396 IV | | miR396* I | | miR397 II | | miR398 II | | miR398 III | | miR398 I | | miR398 IV | | miR398 V | | miR398* I | | miR398* II | | miR398* V | | miR398* IV | | miR398* VI | | miR398* III | | miR408 I | | miR408 II | | miR408 III | | miR444 III | | miR444 II | | miR5072 I | | miR5072 II | | miR5139 | | miR528 I | | miR528 II | | miR529 I | | miR529* I | | miR827 | | | TGACAGAAGAGAGTGAGCAC | | --- | | TTGACAGAAGAGAGTGAGCAC | | TGACAGAAGAGAGTGAGCA | | TGACAGAAGAGAGCGAGCAC | | TTGACAGAAGAGAGCGAGCAC | | TGACAGAAGAGAGTGAGCACG | | GACAGAAGAGAGTGAGCA | | TGACAGAAGAGAGTGAGC | | TGACAGAAGAGAGTGAGCACA | | CTGACAGAAGAGAGTGAGCAC | | GACAGAAGAGAGTGAGCACA | | GCTCACTTCTCTTTCTGTCAGC | | GCTCACTCTCTATCTGTCAGC | | GCTCACTTCTCTCTCTGTCAGC | | TGCTCACTTCTCTTTCTGTCAGC | | GCTCGCTTCTCTTTCTGTCAGC | | GCTCACTCTCTATCTGTCAG | | GCTCACTTCTCTTTCTGTCAG | | TTTGGATTGAAGGGAGCTCTG | | TTTGGATTGAAGGGAGCT | | TTTGGATTGAAGGGAGCTCT | | TTGGATTGAAGGGAGCTCTG | | TTTGGATTGAAGGGAGCTCTGC | | TTTGGATTGAAGGGAGCTC | | TGCCTGGCTCCCTGTATGCCA | | TGCCTGGCTCCCTGAATGCCA | | TCGATAAACCTCTGCATCCAG | | TGGAGAAGCAGGGCACGTGCA | | TGGAGAAGCAGGGCACGTGCT | | TCGGACCAGGCTTCATTCCCC | | TCGGACCAGGCTTCATTCCTC | | TCTCGGACCAGGCTTCATTCC | | TCGGACCAGGCTTCATTCCC | | TTCGGACCAGGCTTCATTCCC | | TCGGACCAGGCTTCAATCCCT | | GGAATGTTGTCTGGCTCGAGG | | GGAATGTTGTCTGGTTCAAGG | | GGAATGTTGTCTGGCTCGGGG | | TGAAGCTGCCAGCATGATCTGA | | TGAAGCTGCCAGCATGATCTG | | TGAAGCTGCCAGCATGATCTGG | | TGAAGCTGCCAGCATGATCTA | | AAGCTGCCAGCATGATCTGA | | ATGAAGCTGCCAGCATGATCTGA | | AGGTCATGCTGTAGTTTCATC | | AGATCATCTGGCAGTTTCATT | | TCGCTTGGTGCAGATCGGGAC | | TCGCTTGGTGCAGATCGGGA | | CCCGCCTTGCACCAAGTGAAT | | TCGCTTGGTGCAGATCGGGACC | | GATCCCGCCTTGCACCAAGTGAAT | | CAGCCAAGGATGACTTGCCGG | | CAGCCAAGGATGACTTGCCGA | | AGCCAAGGATGACTTGCCGAT | | AGCCAAGGATGACTTGCCGG | | TAGCCAAGGATGACTTGCCTACA | | AGCCAAGGATGACTTGCCGGC | | TAGCCAAGGATGAGCTGCCTG | | TAGCCAAGGATGACTTGCCGG | | TGGGCAGTCTCCTTGGCTAGC | | TGGCATCCATTCTTGGCTAAG | | GCATCCATTCTTGGCTAAGTG | | TGATTGAGCCGTGCCAATATC | | TTGAGCCGCGTCAATATCTCC | | TGAGCCGAACCAATATCACTC | | TTGAGCCGTGCCAATATCACG | | AGAATCTTGATGATGCTGCAT | | TTGGACTGAAGGGTGCTCCCT | | CTCCAAAGGGATCGCATTGAT | | TCCAAAGGGATCGCATTGAT | | TCAGTGCAATCCCTTTGGAAT | | TTGGCATTCTGTCCACCTCC | | TGAAGTGTTTGGGGGAACTC | | TGAAGTGTTTGGAGGAACTC | | TCCACAGGCTTTCTTGAACTG | | TTCCACAGCTTTCTTGAACTT | | TCCACAGGCTTTCTTGAACT | | CTTTCCACAGGCTTTCTTGAA | | ATGGTTCAAGAAAGTCCTTGGAAA | | ACAGGCTTTCTTGAACTG | | TTCCACAGCTTTCTTGAACT | | GTTCAAGAAAGTCCTTGGAAA | | TTTCCACAGCTTTCTTGAACT | | TTCCACAGCTTTCTTGAACTG | | GGTCAAGAAAGCTGTGGGAAG | | TTGAGTGCAGCGTTGATGAGC | | TGTGTTCTCAGGTCGCCCCCG | | GTGTTCTCAGGTCGCCCCCGC | | ATGTGTTCTCAGGTCGCCCCCG | | ATGTGTTCTCAGGTCGCCCCC | | GTGTTCTCAGGTCGCCCCCG | | GGGGGCGGACTGGGAACACAT | | GGGGCGGACTGGGAACACATG | | GGGGGCGGACTGGGAACACA | | GGGGCGGACTGGGAACACA | | GGGGGCGGACTGGGAACAC | | GGGGCGGACTGGGAACACAT | | CTGCACTGCCTCTTCCCTGGC | | TGCACTGCCTCTTCCCTGGCT | | TGCACTGCCTCTTCCCTGGC | | TGCAGTTGTTGTCTCAAGCTT | | TGCAGTTGTTGCCTCAAGCTT | | TTCCCCAGCGGAGTCGCCA | | GTCCCCAGCGGAGTCGCCA | | AACCTGGCTCCGATACCA | | TGGAAGGGGCATGCAGAGGAG | | TGGAAGGGGCATGCAGAGGA | | AGAAGAGAGAGAGTACAGCCT | | GCTGTACCCTCTCTCTTCTTC | | TTAGATGACCATCAGCAAACA | | | 26876,73 | | --- | | 2085,19 | | 958,68 | | 629,99 | | 203,39 | | 169,59 | | 146,28 | | 139,28 | | 112,48 | | 90,91 | | 69,35 | | 5650,07 | | 5536,43 | | 1234,33 | | 426,01 | | 177,17 | | 171,92 | | 101,40 | | 80372,02 | | 2111,42 | | 1473,86 | | 1301,93 | | 191,74 | | 104,32 | | 980,24 | | 97,91 | | 368,90 | | 403,29 | | 60,61 | | 20235,35 | | 2782,20 | | 861,35 | | 775,10 | | 616,58 | | 294,30 | | 131,71 | | 119,47 | | 111,89 | | 44576,98 | | 5159,37 | | 2412,72 | | 1064,74 | | 148,03 | | 58,28 | | 406,20 | | 127,63 | | 36249,03 | | 8045,89 | | 692,93 | | 435,34 | | 58,28 | | 20765,10 | | 396,29 | | 307,71 | | 218,54 | | 159,10 | | 146,86 | | 125,88 | | 79,26 | | 243,02 | | 233,11 | | 156,77 | | 782,09 | | 249,43 | | 70,52 | | 64,69 | | 102,57 | | 487,21 | | 1969,22 | | 92,66 | | 171,92 | | 381,72 | | 965,67 | | 189,99 | | 52540,11 | | 1996,03 | | 622,41 | | 460,98 | | 191,74 | | 85,67 | | 82,17 | | 66,44 | | 66,44 | | 58,86 | | 97,32 | | 2930,81 | | 5304,48 | | 446,41 | | 375,31 | | 79,26 | | 59,44 | | 4634,28 | | 499,44 | | 392,21 | | 217,96 | | 109,56 | | 90,33 | | 1307,76 | | 1015,21 | | 379,97 | | 342,68 | | 130,54 | | 565,88 | | 263,42 | | 61,19 | | 1683,66 | | 166,09 | | 850,28 | | 560,64 | | 190,57 | |
