# Supplementary material for: High-Throughput Sequencing of Small RNA Transcriptome Reveals Salt Stress Regulated MicroRNAs in Sugarcane
Source: PLoS One. 2013 Mar 27;8(3):e59423. doi: 10.1371/journal.pone.0059423 (PMC3609749; doi:10.1371/journal.pone.0059423)
Supplement: Table S2 — miRNAs and targets primers sequences for RT-PCR analysis. (DOC) [file pone.0059423.s004.doc]

Table S2: miRNAs and targets primers sequences for RT-PCR analysis.

| **Primers sequences** | | |
| --- | --- | --- |
| **miRNAs** | **Forward( 5’- 3’)** | **RT( 5’- 3’)** |
| miR159 XVI | CGGCGGTTTGGATTGAAGGGA | GTCGTATCCAGTGCAGGGTCCGAGGTATTCGCACTGGATACGACCAGAGC |
| miR166 III | GCGGCGTCGGACCAGGCTTCA | GTCGTATCCAGTGCAGGGTCCGAGGTATTCGCACTGGATACGAGGGGAA |
| miR167 V | GCGGCGGCGTGAAGCTGCCAGCATG | GTCGTATCCAGTGCAGGGTCCGAGGTATTCGCACTGGATACGACTCAGAT |
| miR168 II | CGGCGGTCGCTTGGTGCAGAT | GTCGTATCCAGTGCAGGGTCCGAGGTATTCGCACTGGATACGACGTCCCG |
| miR169 III | CGGCGGCAGCCAAGGATGACT | GTCGTATCCAGTGCAGGGTCCGAGGTATTCGCACTGGATACGACCCGGCA |
| miR396 II | CGGCGGTCCACAGGCTTTCTT | GTCGTATCCAGTGCAGGGTCCGAGGTATTCGCACTGGATACGACCAGTTC |
| miR397 II | CGGCGGTTGAGTGCAGCGTTG | GTCGTATCCAGTGCAGGGTCCGAGGTATTCGCACTGGATACGACGCTCAT |
| miR398 II | CGGCGGTGTGTTCTCAGGTCG | GTCGTATCCAGTGCAGGGTCCGAGGTATTCGCACTGGATACGACCGGGGG |
| miR398* I | CGGCGGGGGGGCGGACTGGGA | GTCGTATCCAGTGCAGGGTCCGAGGTATTCGCACTGGATACGACATGTGT |
| miR156 V | GCGGCGGCGTGACAGAAGAGAGT | GTCGTATCCAGTGCAGGGTCCGAGGTATTCGCACTGGATACGACGTGCTC |
| miR528 I | CGGCGGTGGAAGGGGCATGCA | GTCGTATCCAGTGCAGGGTCCGAGGTATTCGCACTGGATACGACCTCCTC |
| Universal Reverse Primer | GTGCAGGGTCCGAGGT |  |
| **Targets** | **Forward ( 5’- 3’)** | **Reverse ( 5’- 3’)** |
| AGO1 | CGTGGCGGGCATGTG | TGGTCTAGATGGACCTGAAGGAA |
| GRF | AAGCCTACCCGGACTCCAA | AGGCTTTCTTGAACGGTTCTTG |
| LAC | GGCGCCACCATGTCTTG | TGACTGCAGCAATGCTAGCAA |
| GAMyB |  |  |
| Ser/Thr | CCGCGAAGAGCGATGTGTA | GCCCAGACAGCAACTCCAA |
| HAP12 | CTTATGGCTCACCTGCTATTATGC | GGCACTCGAGAGGACGAAAC |
